# Advances in prevention and therapy of neonatal dairy calf diarrhoea: a systematical review with emphasis on colostrum management and fluid therapy

**DOI:** 10.1186/s13028-014-0075-x

**Published:** 2014-11-25

**Authors:** Vanessa Meganck, Geert Hoflack, Geert Opsomer

**Affiliations:** Department of Reproduction, Obstetrics and Herd Health, Faculty of Veterinary Medicine, Ghent University, Salisburylaan 133, 9820 Merelbeke, Belgium; MSD Animal Health, Lynx Binnenhof 5, 1200 Brussels, Belgium

**Keywords:** Neonatal calf diarrhoea, Fluid therapy, Colostrum management

## Abstract

Neonatal calf diarrhoea remains the most common cause of morbidity and mortality in preweaned dairy calves worldwide. This complex disease can be triggered by both infectious and non-infectious causes. The four most important enteropathogens leading to neonatal dairy calf diarrhoea are *Escherichia coli*, rota- and coronavirus, and *Cryptosporidium parvum*. Besides treating diarrhoeic neonatal dairy calves, the veterinarian is the most obvious person to advise the dairy farmer on prevention and treatment of this disease. This review deals with prevention and treatment of neonatal dairy calf diarrhoea focusing on the importance of a good colostrum management and a correct fluid therapy.

## Introduction

Neonatal calf diarrhoea (NCD), defined in this paper as diarrhoea in calves aged 1-month-old or younger is a complex disease that can be triggered by both infectious and non-infectious causes. The prevalence and incidence risk for NCD in dairy cattle herds has recently been reported to be 19.1 and 21.2%, respectively [1,2]. In calves aged <31 days, enteritis is the most common cause of death with a case fatality risk for NCD of 4.9% and a peak probability of dying due to enteritis during the second week of life [1,3]. Enterotoxic *Escherichia coli K99/F5*, rota- and coronavirus, and *Cryptosporidium spp.* (≥85% *C. parvum*) are the 4 most important enteropathogens causing NCD worldwide with rotavirus and *C. parvum* most frequently identified in faecal samples from young calves [2,4-7]. *Escherichia coli K99/F5* typically causes diarrhoea in calves 1–4 days old and the other three cause diarrhoea most often in 1 to 3-week-old calves. Neonatal calf diarrhoea prevalence for *E. coli*, rota- and coronavirus, and *C. parvum* ranges from 2.6-45.1%, 17.7-79.9%, 3.1-21.6% and 27.8-63.0%, respectively [2,5,7-12]. However, the enteropathogens most commonly implicated in NCD outbreaks can also be found in faecal samples from healthy calves, meaning that the presence of a pathogen is not always causative [2,7]. Less common enteropathogens causing NCD are *Salmonella spp.*, attaching and effacing *E. coli* among which enteropathogenic *E. coli*, enterohemorrhagic *E. coli* and Shiga toxin producing *E. coli*, *Clostridium difficile*, *Clostridium perfringens*, and torovirus [2,13-16]. The veterinarian has an important advisory role in the daily prevention and treatment challenge in practice. The aim of this review is therefore to provide a critical analysis of recent literature on prevention and treatment of NCD with emphasis on colostrum management and fluid therapy.

## Search strategy and inclusion and exclusion criteria for references

Web of Science (https://webofknowledge.com/) was searched in September 2014 with ‘calf’ AND ‘neonatal’ AND ‘diarrhoea’ AND ‘fluid therapy’ (46 references) followed by ‘calf’ AND ‘neonatal’ AND ‘diarrhoea’ AND ‘colostrum’ (136 references) as subject-specific terms. Articles published in peer reviewed journals of the past decade (2004-present) were considered to retain reliable and recent advances on prevention and treatment of NCD (93 references). Literature reviews and case reports were not included but were scanned for missed references (190 references). Relevance screening was conducted by the first author withholding only English papers dealing with colostrum management or fluid therapy in conjunction with neonatal diarrhoea in dairy calves (105 references). There were no publication status restrictions.

## Review

### Prevention

Dairy farms with a confirmed NCD diagnosis should consult a veterinarian. Together they should go through the herd anamnesis addressing the young stock management creating a list with possible critical control points. Key questions in this anamnesis are: colostrum management, housing and hygiene, feeding of the calves, possible periods of stress, drugs used, and prevention of immunomodulating infectious diseases (e.g. bovine viral diarrhoea virus or infectious bovine rhinotracheitis virus).

Colostrum management is the most important preventive measure that should be addressed and this will be discussed in further detail later in this review.

In view of the increasing pressure in the veterinary field to lower the use of antimicrobials (AB), using AB as a prophylactic or metaphylactic measure is debatable. Metaphylactic use of AB can only be recommended for a short period on herds actively struggling with *E. coli* diarrhoea problems. Furthermore, calves receiving prophylactic AB in their milk for the first 2 weeks of life have a 28% greater risk for diarrhoea compared with calves receiving no prophylactic AB in their milk [17].

In a Swedish study in 2010 disinfection of single pens between calves was more common in herds suffering from NCD when compared to herds without problems [7]. This can be explained by herds starting with disinfecting procedures if suffering from NCD. Single pens should be disinfected before moving any new calf and not only when an outbreak of NCD occurs.

The control of *C. parvum* can only be achieved by combining a good hygiene management and effective preventive drugs [18-20]. Newborn calves should not be mixed with older calves since the age of the calf is an important risk factor for shedding of *C. parvum* oocysts [18,21-23]. In Europe, halofuginone lactate is the only registered product for prevention and treatment of *C. parvum*. Different studies find a delayed and lower oocyst output peak the first 2 weeks after birth in halofuginone treated groups as compared to placebo groups [21,24-27].

Calves born to dams vaccinated against *E. coli*, rotavirus and coronavirus also shed less *C. parvum* oocysts [6]. This lower shedding of *C. parvum* oocyst is most likely a reflection of a generally higher standard of herd management in these herds, rather than a direct protective effect. A vaccine against *C. parvum* has not been developed yet.

The literature is contradictory or inconclusive concerning ancillary preventive products such as phytopharmaceuticals (e.g. clinoptilitezeolite) and probiotics (e.g. *E. coli* Nissle 1917) [28-30]. The herd veterinarian is advised to consult results of peer-reviewed controlled trials for separate products.

#### Colostrum management

Although the importance of a good colostrum management leading to an adequate passive transfer is undebatable in the prevention of NCD [17,31-33], there are studies showing no significant effect of colostrum feeding routines on the risk of diarrhoea or on the risk of shedding *C. parvum* [4,6]. This lack of a significant effect can be explained by a high number of diarrhoea cases caused by *C. parvum*, for which colostral IgG is less protective [6]. An FPT prevalence <10% is considered as a rational and achievable goal when using a cut-off value of 10 g IgG/l [34]. However, several studies proposed serum IgG concentrations up to 15 g/l as cut-off points for defining failure of passive transfer (FPT) [1,35,36]. Percentages of FPT range from 8.4 to >90.5% depending on the cut-off value, the population studied, and/or the method used to estimate calf serum IgG levels [1,12,17,37-39]. The odds of FPT are higher when there is no on-farm routine screening [38]; therefore, IgG concentration of 48-hours-old calves should be estimated regularly to test the compliance of the colostrum management and is most practical in the field by determining serum total protein using a refractometer.

Colostrum also contains other beneficial constituents in higher concentrations than normal milk: immunologically active leukocytes, fat, protein, fat-soluble vitamins (e.g. retinol, tocopherol, β-carotene), water-soluble vitamins (e.g. niacin, thiamine, riboflavin, vitamin B12, pyridoxal, pyridoxamine, pyridoxine), minerals (e.g. Ca, P, Mg, Na, K, Zn, Fe, Cu, S, Mn) and, non-specific antimicrobial factors (e.g. lactoferrin) [40].

The functional importance of colostral leukocytes is not yet fully understood. Some studies show that they enhance lymphocyte responses to nonspecific mitogens and specific antigens and increase antigen-presenting capacity [41-44], while others suggest that the role of fresh colostral leukocytes may not be as important as once thought [45]. As protection against *C. parvum* is mainly cell-mediated, further research into the importance of colostral leukocytes is warranted.

#### Colostrum quality

##### IgG concentration

Colostral IgG concentration is an important factor that affects whether calves receive sufficient passive immunity from colostrum [46]. Unfortunately, the amount of IgG in maternal colostrum varies dramatically among cows (<1-235 g/l) with 29.4-57.8% of samples that do not reach the desired amount of 50 g IgG/l [34,47-49]. Colostral quality is difficult to estimate by the farmer based on produced volume or appearance of the colostrum. Use of weight at first milking as a screening test to identify bovine colostrum with inadequate IgG concentration is not justified because of the low sensitivity [50]. Somatic cell count, measured after calving, was significantly higher in cows producing colostrum of inferior quality compared with those producing high-quality colostrum [47]. For all these reasons, it is better to measure colostral IgG content indirectly by using a colostrometer (50 g IgG/l = density of 1045) or a brix refractometer (50 g IgG/l =21-22 Brix) or directly by a cow-side immunoassay kit (single line = IgG concentration >50 g IgG/l [50-53]. To measure colostrum quality it should be avoided to use forestripping samples for testing purposes, as these samples may overestimate the IgG concentration [54]. Colostrum collected more than 2 hours after calving significantly lowers the colostral IgG concentration [55,56], probably because of dilutional effects and because colostral Ig diffuse passively into the cow’s systemic circulation if colostrum is not milked out as soon as possible after calving. Colostrum from cows without a dry period has a lower IgG concentration compared with colostrum from cows having a dry period of 28 or 56 days [57]. Pooling of colostrum is not advised since this is a risk factor for FPT [38]. Cows in their 3^rd^ or 4^th^ parity or older usually have significantly higher levels of IgG per liter colostrum than heifers or 2^nd^ parity cows [34,47,48] which is reflected in calves born to heifers having a greater risk for FPT than calves born to multiparous cows [32]. However, this correlation between parity and colostrum IgG concentration is not always evident [36]. Discarding colostrum from heifers is thus not advisable.

Colostrum replacement products have a highly variable performance [49,58-65], and the herd veterinarian is advised to consult results of peer-reviewed controlled trials for separate products. Colostrum supplemented in milk replacer during the first 2 weeks of life (10 g IgG, bid) reduces NCD rates because of the intestinal activity of colostral antibodies and epithelial growth promoting substances [66].

##### Bacteriological quality

Critical control points for bacterial contamination of colostrum are the harvest and storage process. Storing colostrum at ambient temperatures results in a significant increase of bacteria [67]. Fresh colostrum can be stored up to 96 hours at 4°C when potassium sorbate is added as a preservative [67]. Industry recommendations for bacterial load are limited to 100,000 colony forming units (cfu)/ml. Depending on the time of sampling or used storage method, 0-43% of colostrum samples exceed the maximum advised bacterial count of 100,000 cfu/ml [48,67].

Pasteurizing colostrum (60°C, 60 min) can reduce bacterial load while the viscosity and the colostral IgG concentration remain within acceptable limits for feeding [68-72]. Higher-quality batches of colostrum suffer a significantly greater magnitude of loss of IgG as compared with lower- or intermediate- quality batches of colostrum [68,71]. Calves fed pasteurized colostrum had significantly higher serum IgG concentrations, serum protein values, and/or a greater apparent efficiency of absorption, compared with calves fed unpasteurized colostrum [46,72-75], probably explained by bacteria that bind free IgG in the gut lumen and block uptake and transport of IgG molecules across intestinal epithelial cells. Moreover, calves fed pasteurized colostrum had a significantly decreased risk for treatment for diarrhoea [46].

#### Colostrum feeding practices

Calves allowed to nurse their dam have higher odds of FPT than calves separated from their dam within 3 hours of birth [37,38]. When allowed to nurse, calves drink too late and too little colostrum. Feeding calves as much colostrum as they want by nipple bottle within 1–4 hours after birth and at 12 hours of age substantially reduces the probability of FPT [34,37,76]. Bottle fed calves that do not ingest colostrum voluntarily, should be tube fed [34,76]. Feeding at least 150 to 200 g of colostral IgG is required for adequate passive transfer of colostral IgG when colostrum is administered once by oesophageal intubation <2 hours after birth [36]. To estimate the exact amount of IgG given to a calf, colostrum quality should be measured as explained higher: if colostrum contains 50 g IgG/L, feeding 4 L of colostrum suffices to provide 200 g IgG to the calf. There is no added benefit in feeding 4 L of colostrum compared to 3 L when colostrum of comparable quality is fed once using an oesophageal tube [77]. Larger IgG intakes are required by calves being fed >2 hours after birth [36]. Hand feeding colostrum >4 hours after birth is a risk factor for FPT [38]. A slight delay in the increase of serum Ig concentration was obvious in calves receiving 4 L of colostrum by oesophageal intubation compared with bottle-fed calves receiving 2 L. However, calves receiving colostrum by oesophageal intubation reached significantly higher Ig concentrations compared with bottle-fed calves [78]. Because of the difference in administered volume (4 L versus 2 L) between the two methods (oesophageal intubation versus bottle-fed), it can only be concluded from this study that an appropriate use of an oesophageal tube to feed 4 L colostrum is a safe and reliable method for an adequate passive immune transfer in healthy newborn calves. This study does not prove colostrum fed by oesophageal intubation to be better than bottle-feeding colostrum. Further research is needed to reveal if the rapid passage of Ig from the reticulorumen to the abomasum also occurs in weak born calves. Moreover, tube feeding can cause moderate depression or a more difficult adaptation to feeding with a nipple bucket [78].

Calves cared for by female workers are less likely to develop FPT [37], probably because women are more patient with newborn calves that refuse to suckle. The odds of FPT are higher for calves experiencing dystocia [32] which can be explained by drinking too little colostrum and/or a lower apparent efficiency of absorption.

### Treatment

#### *Fluid therapy*

Diarrhoea leads to dehydration, acidosis, electrolyte imbalance, and hypoglycemia, all of which should be addressed by a well-executed fluid therapy management [31,79,80].

Metabolic acidosis in diarrhoeic calves arises not only from fecal bicarbonate loss and hyper-L-lactatemia, but mainly from a hyper-D-lactatemia. Hyper-L-lactatemia is a result of dehydration and decreased tissue perfusion. Hyper-D-lactatemia is caused by absorption of D-lactic acid produced in the gastro-intestinal tract by fermentation of malabsorbed carbohydrates [81,82]. Variations in behaviour, posture and palpebral reflex are more closely correlated with elevations of serum D-lactate concentrations than with decreases in base excess (BE) [79,83,84]. However, BE values can be estimated based on posture, behaviour, and palpebral reflex (Table 1) with calves aged ≥7 days having lower BE values than younger calves [31,84,85]. Impairment of a good sucking reflex is more related to the degree of dehydration than it reflects acidosis [79,83,86]. There is even no obvious correlation between the serum levels of D-lactate and dehydration [82]. Remarkably, ability to stand was maintained in quite some calves despite BE values below −20 mmol/l, demonstrating the risk of undercorrection when based on this ability [85].

**Table 1:** **Guide to assessing the degree of acidosis based on posture, behaviour and palpebral reflex of the calf (adapted from** [85]**)**

	**Category**	**BE (mmol/l)**
**Posture**	Calf standing up by itself	−2.2
	Calf standing up after encouragement	0.0
	Standing steadily after lifting	2.8
	Standing unsteadily, able to correct position if forced	−11.7
	Standing unsteadily, unable to correct position if forced	−20.6
	Unable to stand, sternal recumbency	−20.9
	Unable to stand, lateral recumbency	−25.4
**Behaviour**	Adequate reaction to acoustic and optical stimuli, very bright and alert	2.5
	Adequate reaction to acoustic and optical stimuli	1.8
	Delayed reaction to acoustic and optical stimuli	−14.6
	Calf reacts only to painful stimuli	−21.0
	No reaction to painful stimuli	−25.4
**Palpebral reflex**	Eyelids are closed immediately and fully	0.7
	Eyelids are closed immediately but not fully	−7.6
	Eyelids are closed with delay and not fully	−19.9
	Eyelids are not closed at all	−24.3

High potassium levels are more closely correlated to dehydration than to parameters indicative of metabolic acidosis [87,88]. Therefore, K^+^ levels should be decreased to normal by correction of the acidosis and hypoglycaemia but more importantly by improving tissue perfusion.

##### Oral rehydration therapy

Administration of an oral rehydration solution (ORS) should be commenced as soon as diarrhoea starts and should be continued as long as the calf has diarrhoea.

The osmolality of an ORS is determined primarily by the concentrations of sodium and glucose. Calves fed hyperosmotic ORS solutions (600–717 mOsm/l) (HORS) have a slower abomasal emptying rate compared with calves fed iso-osmotic ORS solutions (300–360 mOsm/l) (IORS). This slower emptying rate increases the risk for bloat or abomasitis and produces a slower rate of plasma volume expansion [80,89,90]. Unless previously assumed, the volume present in the reticulorumen of healthy calves after intubation appears to be predominantly due to reflux from the abomasum, rather than spillage from incomplete closure of the oesophageal groove. The calculated change in abomasal volume of intubated calves approximated that one of calves that suckled their ORS [80]. In contrast, the delivery of HORS to the small intestine is slower after intubation indicating a different effect of intubation on coordinating motility between the reticulorumen, abomasum and duodenum than does suckling [80]. These effects taken together, suckling an IORS provides the fastest rate of solution delivery to the small intestine and a slightly faster rate of plasma volume expansion than does suckling or oesophageal intubation of a HORS. However, suckling or oesophageal intubation of a HORS provides the most appropriate oral solution for treating hypoglycaemic calves, because the HORS produces a larger and more sustained increase in plasma glucose concentration [80].

Alkalinizing agents commonly used in commercial ORS are bicarbonate and bicarbonate precursors, mainly acetate, propionate, citrate and phosphate. The higher assumed alkalinisation of ORS products containing bicarbonate (BORS) or citrate (CORS) when compared to solutions containing other bicarbonate precursors is confirmed by most recent studies in absolute numbers, but never significantly different [90-93]. High SID-fluids (≥79 mmol/l) are recommended to treat acidosis because the SID value of an ORS determines the degree of abomasal and serum alkalinisation [91,94]. Prolongation of the *in vivo* clotting time is more likely to occur when larger volumes of an ORS in cow’s milk are fed, because luminal pH will initially be greater and remain greater for a longer period of time [90,95]. From these studies it can be carefully concluded that feeding a BORS or CORS does not affect milk clotting *in vivo* and disagrees with general recommendations to not feed BORS or CORS concurrently with or short before/after cow’s milk to calves. The SID value and fed amount of an ORS seem to play a more important role, both in correcting dehydration and metabolic acidosis [91,94].

It should be noted that most of the research discussed above was executed in a small number of healthy, non-diarrhoeic calves. Milk clotting, abomasal luminal pH and abomasal emptying rates could differ between diarrhoeic and non-diarrhoeic calves.

##### Intravenous fluid therapy

Intravenous (IV) fluid therapy should be implemented if the calf is severely dehydrated (>8%), depressed, has a weakened/absent suckle reflex or suffers from a dilated abomasum and/or intestinal hypomotility. The fluids should always be warmed e.g. by adding a coil to the IV fluid line and placing this in a bucket of hot water so that the calf does not need to use extra energy to bring the given fluid to body temperature. To sustain improved clinical status, an IV fluid therapy needs to be followed by continued ORS therapy [31,96]. Both the jugular as the auricular veins can be catheterized for IV rehydration, but by using the ear vein, larger quantities of fluid can be given during a longer period of time in an on farm setting.

Bicarbonate requirements for IV correction of acidosis are still being calculated as follows: bicarbonate (g) = body weight (kg) × BE (mmol/l) × 0.6 (l/kg) × 0.084 (g/mmol) [86,96]. When using this formula, metabolic acidosis was not corrected in more than half of the calves and the risk of failure to correct acidosis increased with D-lactate concentrations [86]. Calves with distinct changes in posture and demeanour thus seem to need higher doses of bicarbonate than calculated with the factor of 0.6 in the formula mentioned. However, in this study no follow-up therapy with ORS was offered, which could explain calves becoming acidotic again after 24 h. Other studies reporting treatment failures had a more severe metabolic acidosis before treatment compared with successfully treated calves [31,96]. The formula mentioned above should only be used for estimation of buffer required for correction of incurred losses and overdosing seems more desirable than underdosing [86]. Bicarbonate can be given IV as an isotonic (IBS) or hypertonic solution (HBS). In the field it is more practical to use a HBS compared with an IBS. Rapid IV administration of an 8.4% bicarbonate formulation at 5–10 ml/kg provided an effective and safe method to improve acid–base abnormalities [31,96-98]. There were no direct indications in these studies of potential adverse effects related to electrolyte concentrations, oxygen-haemoglobin dissociation curve, hypercapnia or paradoxical intracellular acidosis, as previously assumed. It can be concluded from these studies that HBS can be safely used if the speed of an IV administration does not exceed 1.25 ml/kg/min in calves not suffering from respiratory problems.

Hypoglycaemia is best addressed by giving 150 mg glucose/kg. Larger quantities of glucose should be avoided because of the risk of glycosuria. Calves that are cachectic and reluctant to drink can also be given a constant glucose infusion using the ear vein approach as discussed earlier.

#### Other important treatment measures

Routine use of AB in diarrhoeic calves cannot be recommended due to increased levels of antibiotic resistance. However, systemically ill calves (depression, anorexia, fever) often suffer from *E. coli* septicaemia and thus parenteral Gram-negative-spectrum AB are advised to treat these calves. Antimicrobial susceptibility testing methods should be performed on the herd level at a regular basis [99,100]. As mentioned, halofuginone lactate is the only registered product for prevention and treatment of *C. parvum* in Europe. In 2009 a meta-analysis for the effects of therapeutic halofuginone treatment was conducted [21]. Their results were considered as uninterpretable because of a lack of sufficient data. Azithromycin (1,500 mg/d, 7 days, per oral (PO) and lasalocid (8 mg/kg, sid, 3 days, PO) reduce oocyst shedding [101,102]. Therapeutic use of nitazoxanide (15 mg/kg, bid, PO, 10 days) did not improve the clinical appearance, nor the intensity of oocyst excretion [103]. In contrast, calves of the therapeutic group showed a longer diarrhoeic episode compared to the untreated control group. Antiviral drugs to treat rota- and coronavirus are not commercially available. Non-steroidal anti-inflammatory drugs (NSAID) like meloxicam (0.5 mg/kg, one-shot, SC) improve appetite and growth rate, probably because NCD is accompanied by malaise and gastrointestinal discomfort [39]. Despite their widespread use, there is no valid recent research available to recommend products reducing intestinal motility (e.g. hyoscine N-butylbromide).

The literature is contradictory or inconclusive concerning other ancillary treatments such as phytopharmaceuticals and probiotics [104,105]. For example, the number of days having diarrhoea, severity of diarrhoea and mortality rates were similar between an oral treatment with neomycin or dried oregano leaves [105]. However, a negative control group receiving no treatment was not included and they also defined calves older than 4 days with diarrhoea to suffer from colibacillosis. *Lactobacillus rhamnosus* GG did not affect the outcome of therapy in diarrhoeic calves [104]. The herd veterinarian is advised to consult results of peer-reviewed controlled trials for separate products.

Overall, the herd veterinarian should focus on the prevention of NCD.

## Conclusions

Neonatal calf diarrhoea is a complex disease with a high morbidity and mortality in dairy cattle herds. Colostrum management is the most important preventive measure but is often neglected. Also, in the therapy management a good fluid therapy is indispensable. However, mistakes are often made. The herd veterinarian should be up-to-date when it comes to prevention and therapy of NCD. He or she is the most obvious person to communicate these items to the dairy farmer.

## Abbreviations

BORS: Bicarbonate containing oral rehydration solution
cfu: Colony forming units
*C. parvum*: *Cryptosporidium parvum*
CORS: Citrate containing oral rehydration solution
*E. coli*: *Escherichia coli*
FPT: Failure of passive transfer
HBS: Hypertonic bicarbonate solution for intravenous use
IBS: Isotonic bicarbonate solution for intravenous use
IgG: Immunoglobulin G
IV: Intravenous
NCD: Neonatal calf diarrhoea
ORS: Oral rehydration solution
PO: Per oral
SID: Strong ion difference
